# Supplementary material for: Effect of caffeine citrate on diaphragmatic electrical activity in pre-term newborns
Source: PLoS One. 2025 Apr 11;20(4):e0320992. doi: 10.1371/journal.pone.0320992 (PMC11990644; doi:10.1371/journal.pone.0320992)
Supplement: Attachment — Submitted filename: Registration on WHO.docx [file pone.0320992.s001.docx]

I would like to inform you that the research has been registered with the Brazilian Registry of Clinical Trials (an organization approved by the WHO). The necessary documents have been submitted, and we are awaiting approval.

Registration number #15507

Thank you for your understanding.

Dr Tatiana Zidan
