# Supplementary material for: Effect of caffeine citrate on diaphragmatic electrical activity in pre-term newborns
Source: PLoS One. 2025 Apr 11;20(4):e0320992. doi: 10.1371/journal.pone.0320992 (PMC11990644; doi:10.1371/journal.pone.0320992)
Supplement: Attachment — Submitted filename: Response to Reviewers.docx [file pone.0320992.s002.docx]

Dear Editor and Reviewers,

We greatly appreciate all the considerations and suggestions regarding our study. Your observations were very important and added significant value to the article.

Here are our responses and the modifications made according to the suggestions.

Editor#:

1. Please ensure that your manuscript meets PLOS ONE's style requirem

ents, including those for file naming. The PLOS ONE style templates can be found at 
https://journals.plos.org/plosone/s/file?id=wjVg/PLOSOne_formatting_sample_main_body.pdf and 
https://journals.plos.org/plosone/s/file?id=ba62/PLOSOne_formatting_sample_title_authors_affiliations.pdf

We have revised the manuscript in accordance with the PLOS ONE guidelines.

2. Please provide additional details regarding participant consent. In the ethics statement in the Methods and online submission information, please ensure that you have specified (1) whether consent was informed and (2) what type you obtained (for instance, written or verbal, and if verbal, how it was documented and witnessed). If your study included minors, state whether you obtained consent from parents or guardians. If the need for consent was waived by the ethics committee, please include this information.

The information about the consent form is included in the text, under Materials and Methods, as follows:

*“Data were collected after obtaining informed consent (verbal and written) from parents”.*

3. We note that you have a patent relating to material pertinent to this article. Please provide an amended statement of Competing Interests to declare this patent (with details including name and number), along with any other relevant declarations relating to employment, consultancy, patents, products in development or modified products etc. Please confirm that this does not alter your adherence to all PLOS ONE policies on sharing data and materials, as detailed online in our guide for authors http://journals.plos.org/plosone/s/competing-interests by including the following statement: ""This does not alter our adherence to  PLOS ONE policies on sharing data and materials.” If there are restrictions on sharing of data and/or materials, please state these. Please note that we cannot proceed with consideration of your article until this information has been declared.

The study was developed and conducted at the Hospital Israelita Albert Einstein in São Paulo, and none of the authors involved have any conflicts of interest related to patents. The analysis of the collected data was performed in Toronto by Dr. Beck's team. We can confirm that Dr. Beck's participation in the study did not involve any conflicts of interest.

This is Dr.Beck´s response:

This is my disclosure statement (in red) and the confirmation PLOSone is asking for. My disclosure statement cannot be modified, it has been approved by the University of Toronto, by a committee on Conflict of Interest.

The Edi catheter is commercially available, so there is no off-label use to declare.

I do receive royalty for catheter sales, but this is included in mt statement.

"Dr. Beck has made inventions related to neural control of mechanical ventilation that are patented. The patents are assigned to the academic institution(s) where inventions were made. The license for these patents belongs to Maquet Critical Care.  Future commercial uses of this technology may provide financial benefit to Dr. Beck through royalties. Dr Beck owns 50% of Neurovent Research Inc (NVR).  NVR is a research and development company that builds the equipment and catheters for research studies.  NVR has a consulting agreement with Maquet Critical Care. "

I confirm that this does not alter my adherence to all PLOS ONE policies on sharing data and materials.

4. Please amend either the abstract on the online submission form (via Edit Submission) or the abstract in the manuscript so that they are identical.

We used the same abstract in the online submission and in the manuscript.

5. Please include captions for your Supporting Information files at the end of your manuscript, and update any in-text citations to match accordingly.

We have made corrections to the supporting information in accordance with the guidelines.

Reviwer 1#:

1. The fact that the sample size is a convenience sample makes it less powerful - it needs to be highlighted. I wonder why the authors could not have planned a presumed difference in findings before and after caffeine for a single primary outcome and calculation could have ben done. it would have increased the power of the study

The choice of a convenience sample was made because we were unable to conduct an appropriate sample size calculation, as we did not find any literature publications using the variables we employed in this study for such calculation. This was emphasized in the paper for the reader's better understanding.

*“The use of a convenience sample diminishes its power; however, we did not find any literature reference using the same variables for sample size calculation. On the other hand, since we found a significant difference in pre- and post-caffeine use in diaphragm electrical activity, we did not commit a type 2 error”.*

2. Where does the funding for the Edi probe (NAVA) probe come from when it is used for spontaneously breathing / those who did not require any supports? I am curious to know

The NAVA probes were acquired through a research grant from another study (FAPESP grant process number 2013/12499-0). We chose to acknowledge FAPESP (Fundação de Amparo à Pesquisa do Estado de São Paulo) in the acknowledgments and funding section.

3. The figures 1,2 are quite redundant-either the study flow diagram can be expanded to include the primary outcome or the figure can be dropped as such
   the figure 2 also does not add any corroborative information to the methodology

Thank you for the suggestion. We have chosen to exclude Figure 1 and include the information in the text of the manuscript.

*“Out of 78 eligible patients, 42 were excluded (4 major malformations, 18 refusal of consent, 7 data recording issues, 1 severe asphyxia and 12 concurrent use of the system), resulting in 36 included patients”.*

4. Please explain why the signals could not be have been measured and recorded for analysis for a longer duration (like midway between 2 doses) or when the trough level is expected (before the next scheduled dose)?
   the probe should have been in situ or was it removed every time after measurement at 60 min post dose?

The purpose of our study was to record the electrical activity of the diaphragm based on caffeine metabolism. It is kwown that the peak effect of caffeine citrate typically occurs about 30 to 60 minutes after intravenous or oral administration. This is the period when the concentration of caffeine in the blood reaches its highest point, providing the maximum stimulating effect. Based on these facts, the proposed methodology was to obtain the diaphragmatic electrical activity before drug administration and after its peak serum concentration.

Newborns who required an orogastric tube for feeding or newborns who were being ventilated using NAVA mode kept the probe in place. However, newborns who were able to receive oral feeding had the tube removed after measurements were taken. The behavior of caffeine levels and its impact on diaphragmatic electrical activity over time is valuable information and should be the subject of further studies. In our study, this was not possible because not all patients retained the probe after the caffeine loading dose.

5. Table 1 can be better formatted- the max and min need not have a separate column only for 2 variables

We appreciate the suggestion. The table has been formatted.

6. Other details of the respiratory support like how many days of CPAP/NIV/IMV would have more relevance than the proportion of neonates needing each alone
   also clinically relevant outcomes like BPD- to know the sickness levels of the babies- effect of caffeine and diaphragmatic activity would depend on secondary causes of apnea too.

As the aim of our study was to evaluate diaphragmatic electrical activity after administration of the caffeine loading dose, most patients were within the first 48 hours of life. Therefore, it was not yet possible to correlate clinical outcomes such as ventilation time and diagnoses like bronchopulmonary dysplasia. We chose not to include further clinical information to avoid confusing the readers.

Reviwer 2#:

1. There are paragraphs of text that are duplicated in the text, as well as spelling errors (e.g. diaphrgm in the keywords)

Thank you for your observations. Errors have been corrected.

2. The data is fully available on Google Drive. I would have like more information how this would be accesible.

The analysis of diaphragmatic electrical activity data was performed by Dr. Jeniffer Beck's team at the University of Toronto, Canada. All communication was conducted through Google Drive, and if the editorial board of Plos One is interested, access can be granted through the following link:

https://figshare.com/s/2ef5c27321b414529c57

3. The abstract line 37-39 is a very general statement about significance. What I would like to read is what the clinical effect is, not just if it is significant.

Thank you for your observation. We have changed as following.

*“Results showed significant increases in all parameters used to determine the inspiratory phases of respiratory cycles post-caffeine administration (p < 0.001).”*

4. Abstract line 41, you mention 60-120 minutes after loading dose, but measurements were taken until 60min after the loading dose? (line 78) I believe it has something to do with the start of the recording (line 144), but it is confusing.

Thank you for your observation. We agreed and have changed as following.

*“The study concludes that caffeine administration significantly enhances diaphragmatic electrical activity in preterm newborns.”*

5. The authors mention the beneficial effect of caffeine and the fact that it increases diaphragmatic electrical activity (lines 65, reference 12). In line 76 the authors mention other studies investigating the electrical activity, I presume reference 12 should be added to the list as well? Taken together this study does not add significant new data to the already existing amount of data regarding electrical activity after caffeine. It would certainly strengthen the study design to include a much larger group of participants who are (especially) followed for a longer duration. As authors rightfully mention the the discussion. This would really expend the current knowledge about caffeine.

The statement made in line 65 is incorrect and was well observed.

The cited study (reference 12) is an old study conducted on 6 adult patients that evaluated the diaphragm's response to caffeine through transdiaphragmatic pressure measurements obtained via esophageal catheters. According to this study, there was an increase in diaphragm contractility after oral administration of caffeine, and the electrical activity of the diaphragm was not evaluated.

Reference 15 is a study that evaluated diaphragmatic activity through transcutaneous electromyography, a different method from the one used in our study, which evaluated the electrical activity of the diaphragm through the NAVA catheter.

Reference 16 is a study that used the same methodology to obtain diaphragmatic electrical activity, but the main objective was to evaluate the occurrence of apneas after caffeine administration. A limitation of this study was the number of newborns studied, which was much smaller compared to our study.

Reference 17 is a study that used transcutaneous electromyography to evaluate the electrical activity of the diaphragm and only assessed newborns who were on mechanical ventilation.

6. One of my main concerns is the patient groups. It includes many patients on mechanical ventilation, who are often sedated. Also how does one detect apnea's when on mechanical ventilation? As usually there is a set backup breathing frequency? Also many patients are included very early on after birth, where as many patients will develop apneu's within the first days after birth when spontaniously breathing. Suggesting a selection bias in this cohort.

Newborns in our neonatal ICU, as well as in most ICUs, are not sedated when they are on mechanical ventilation. This allows them to maintain spontaneous breathing. None of the patients in our study were sedated. For further clarification for the reader, this comment was added to the text.

*“Respiratory support was defined based on clinical parameters, with no changes between baseline and post-caffeine measurements. None of the pacients were sedated”.*

Our study did not use clinical parameters to detect apneas during the monitoring of diaphragm activity. Respiratory pauses were evaluated through the absence of Edi for 5-10, 11-19, or more than 19 seconds. For this reason, there was no interference from mechanical ventilation in the newborns studied.

We do not agree that there is selection bias in the patients who used caffeine in this study because the inclusion criteria were based on protocols recommended in the literature. The objective of the study was not to select patients with apnea but to evaluate Edi secondary to the use of caffeine.

7. Why are patient with HFO ventilation excluded?

We were not sure if the Edi signal would be adequately transmitted, as there is chest vibration in newborns ventilated with high-frequency ventilation. In any case, there were no eligible cases for the study that used this ventilation mode during data collection.

8. Statistics are mostly based on normality as is suggested by the representation as mean +- SD. In these small populations I think that a non-normal distribution is perhaps a better choice?

As we described in Statistics, parameter distributions were studied using histograms and boxplots, and generalized estimating equation models were applied for the inferential analysis considering the correlation between measurements from the same newborn before and after caffeine administration. Normal or gamma distributions with a logarithmic link function were used, and the distribution that showed the best performance according to the QIC goodness-of-fit results was chosen.

9. Line 192: sample calculation was not performed in my opinion, authors did use "sampling based on convenience" or "convenience sampling".

The choice of a convenience sample was made because we were unable to conduct an appropriate sample size calculation, as we did not find any literature publications using the variables we employed in this study for such calculation. This was emphasized in the paper for the reader's better understanding.

*“The use of a convenience sample diminishes its power; however, we did not find any literature reference using the same variables for sample size calculation. On the other hand, since we found a significant difference in pre- and post-caffeine use in diaphragm electrical activity, we did not commit a type 2 error”.*

10. Line 257: I am unsure what the relationship is between de EDI values and the statement in this line. "These data" suggest that the EDI data is important for this statement, which it is not.

We agree that the statement is not clear because our data do not allow us to relate Edi to serum caffeine levels. To avoid errors in conclusions, we chose to exclude this sentence.

11. Paragraph from line 292 misses a conclusion of how these results should be interpreted in light of the current evidence.

We agree that the paragraph needs a conclusion. We added it to the text.

*“The comparison between the two studies shows a greater increase in Edi using a more refined methodology”.*

12. Line 298, this section should also adress the question whether serum caffeine levels correlate with the clinical effect of caffeine, known in literature.

We agree with the observation and add data from the literature to the text.

*“ Although we could not make this correlation, the literature shows that the standard doses commonly used provide expected clinical effects such as: reduction in apnea episodes and reduction in extubation failures, without increasing the risk of neurodevelopmental disabilities”.*

13. Line 309, in this section authors discuss the negative effect of mechanical ventilation on diaphragm function. This conclusion leads to a limition of this study (the fact that many patients were on MV) which should be discussed.

In our study we were unable to correlate the negative effects of mechanical ventilation on Edi as few patients were in this group (only 13.9%). If there was any effect it was probably small.

*“In our study we could not correlate the ventilatory mode or lack of support with diaphragmatic electrical activity as few patients were on mechanical ventialtion (only 13,9%).”*

Reviwer 3#:

1.I have one issue regarding the required “research integrity”, and this is the registration in a clinical trials registry.

The study was approved by the Ethics and Research Committee of our institution. This Committee is certified by AAHRPP (Association for the Accreditation of Human Research Protection Programs). Therefore, the ethical approval of our studies is approved by the highest possible criteria.

The study was also registered on Plataforma Brasil before it began.

Plataforma Brasil is a national and unified database of research records involving human beings for the entire CEP (Ethics and Research Committee) system. It allows research to be monitored at its different stages - from submission to final approval by CEP, when necessary - including monitoring the field phase, sending partial reports and final research reports (when completed). The system also allows documents to be presented digitally, also providing society with access to public data on all approved research. Through the Internet, it is possible for everyone involved to access, through a shared environment, information together, significantly reducing the processing time of projects throughout the CEP system.

The study was not submitted to Clinical Trials as it was not an interventional study.

2. Regarding required data availability: The statement “Data is avaiable on Google Drive” does not specifically tell how to access data– please add a link or equal, unless you decided to do so after acceptance of the manuscript.

The analysis of diaphragmatic electrical activity data was performed by Dr. Jeniffer Beck's team at the University of Toronto, Canada. All communication was conducted through Google Drive, and if the editorial board of Plos One is interested, access can be granted through the following link:

https://figshare.com/s/2ef5c27321b414529c57

All other modifications suggested by Reviewer 3 have been made, with a single exception regarding the abstract.
