# Supplementary material for: Effect of caffeine citrate on diaphragmatic electrical activity in pre-term newborns
Source: PLoS One. 2025 Apr 11;20(4):e0320992. doi: 10.1371/journal.pone.0320992 (PMC11990644; doi:10.1371/journal.pone.0320992)
Supplement: Attachment — Submitted filename: Response to Reviewers 2.docx [file pone.0320992.s003.docx]

Dear Editor and Reviewers,

We greatly appreciate all the considerations and suggestions regarding our study. Your observations were very important and added significant value to the article.

Here are our responses and the modifications made according to the suggestions.

**Additional Editor Comments:**

1. Spelling mistakes should be corrected with the support of a native English-speaking advisor.

In accordance with the suggestion, we requested that the article be reviewed by American Journal Experts (AJE). The paper was as edited for proper English language, grammar, punctuation, spelling, and overall style. The certificate is attached.

2. There should be harmony between the abstract and the tables in the article, spelling mistakes should be corrected.

The abstract has been corrected, as well as spelling errors.

3. 68% increase in peak inspiratory Edi”, according to Table 1, the maximum inspiratory Edi increased from 10.35 to 12.17, which corresponds to an increase of only 17.6%. Please explain.

Thank you for your comment. Edi inspiratory peak data was not in the table and so the information about the increase seemed incorrect. We add this data to the table.

4. The reference list should be corrected according to the spelling rules.

The reference list has been corrected.

5. It is indicated that it was approved by the ethics committee. The full name of the ethics committee and the approval number, if available, should be included.

We included the full name of the ethics committee and the approval number.

6. In lines 245,246, the conclusion of the study alone gives classical information and it would be good to combine the sentence with the sentence in 247 and 248.

We chose not to modify the conclusion because the objective of our study was to evaluate the electrical activity of the diaphragm and, therefore, our main conclusion was to demonstrate its increase after the caffeine loading dose.

**Reviewer #3**: The authors have answered and addressed many of the questions and issues that arose during the first round of reviews, and I would like to thank them for their effort to improve their manuscript.

My main concern was the fact that approval from the Brazilian Registry of Clinical Trials was sought, but obviously not obtained. The argument that registration with ClinicalTrials.gov was not performed because “it was not an interventional study” is not valid, because ClinicalTrials.gov explicitly invites observational studies “in which researchers simply collect information”. Therefore, I am interested what the answer from the Brazilian Registry of Clinical Trials actually was?

The manuscript was approved by the Brazilian Clinical Trials Registry (REBEC), which is the body approved by the World Health Organization. Approval of the manuscript was registered with number RBR-4x9j9hq. Access address: https://ensaiosclinicos.gov.br/rg/RBR-4x9j9hq

While many of the implemented changes have helped improving the presentation of the manuscript, there are still some changes and improvements required:
1) Introduction, page 3, line 52: Please be consistent when using abbreviations such as AOP.

The abbreviation was removed.

2) Patient population, page 6, line 115: Please change “pacients” to “patients”.

We changed to patients.

3) Table 1: There are differences in the presented mean birth weight between the abstract and the table – please check this carefully and correct it appropriately.

We corrected mean birth weight.

4) Results, page 10, line 221: I suggest changing “age of data collection” to “age at data collection”.

We changed to “age at data collection”.

5) Table 2 and throughout: Please be consistent with the use of dots instead of commas for decimal numbers.

ok
6) Discussion, page 12, line 245: Please change “demonstreted” to “demonstrated”.

We changed to demonstrated.

7) Discussion, page 13, line 280: Where does the “68% increase in inspiratory peak Edi” come from? According to Table 1, the maximum inspiratory Edi increased from 10.35 to 12.17, which only corresponds to an increase of 17.6%. Please explain.

Thank you for your comment. Edi inspiratory peak data was not in the table and so the information about the increase seemed incorrect. We add this data to the table.

8) Discussion, page 13, lines 291-292: I suggest changing “… in the same moment that other routine testes of newborns” to “… together with other routine blood tests”.

Thank you for your comment. We have changed.

9) Discussion, page 14, lines 294-295: The information about “without increasing the risk of neurodevelopmental disabilities” seems not relevant here and could be omitted.

It was done according to the comment.

10) Discussion, page 14, line 308: Please change “ventialtion” to “ventilation”.

We have corrected.
11) The reference list has still not been comprehensively revised and corrected, e.g. for reference 5 “Jonhson” is still incorrect. Furthermore, there are still different styles of referencing used, such as in references 28 and 31.

The reference list has been corrected.
